# ST-Segment Elevation Myocardial Infarction Related to Potential Spontaneous Coronary Thrombosis in Pheochromocytoma Crisis

**DOI:** 10.3389/fendo.2020.00140

**Published:** 2020-03-18

**Authors:** Fei Chen, Mingxia Zheng, Xi Li, Yong Peng, Mao Chen

**Affiliations:** Department of Cardiology, West China Hospital, Sichuan University, Chengdu, China

**Keywords:** myocardial infarction, spontaneous coronary thrombosis, infarct-related coronary artery, hypercoagulability, pheochromocytoma

## Abstract

Pheochromocytoma crisis is a rare and possibly fatal emergency. Hypersecreted catecholamines may result in myocardial injury via its direct toxic effect on cardiomyocytes or mediating vasoconstriction which will reduce coronary blood flow in this special setting. Interestingly, several case studies have reported the occurrence of ST-segment elevation myocardial infarction in patients with pheochromocytoma crisis. However, no one found the angiographic evidence of occlusive thrombus in the infarct-related coronary artery. Additionally, pheochromocytoma can induce hypercoagulability and promote thrombosis, but spontaneous coronary thrombosis has never been reported in this condition. Here, we report an unusual case of pheochromocytoma crisis presenting with STEMI due to spontaneous coronary thrombosis.

## Introduction

Pheochromocytoma crisis is a rare and possibly fatal emergency. It often involves the cardiovascular system, manifesting as severe hypertension, circulatory shock, arrhythmias, myocardial injury, or Takotsubo cardiomyopathy (1–3). The mechanisms for myocardial injury in this special condition may be mediated by excessively secreted catecholamines which have a direct toxic effect on cardiomyocytes and can reduce coronary blood flow via vasoconstriction (3). Interestingly, several case studies have reported the occurrence of ST-segment elevation myocardial infarction (STEMI) in patients with pheochromocytoma crisis (4). However, no studies found the angiographic evidence of occlusive thrombus in the infarct-related coronary artery of these patients, which may be the most robust evidence of myocardial infarction except histopathological findings. Additionally, pheochromocytoma can induce hypercoagulability and promote thrombosis (5), and some researchers have found the phenomenon of spontaneous thrombosis in patients with pheochromocytoma (6). Nevertheless, this phenomenon occurring in the coronary artery has never been reported. Here, we report an unusual case of pheochromocytoma crisis presenting with STEMI confirmed by the angiographic evidence of occlusive thrombus due to potential spontaneous coronary thrombosis.

## Case Report

**Figure 1:**
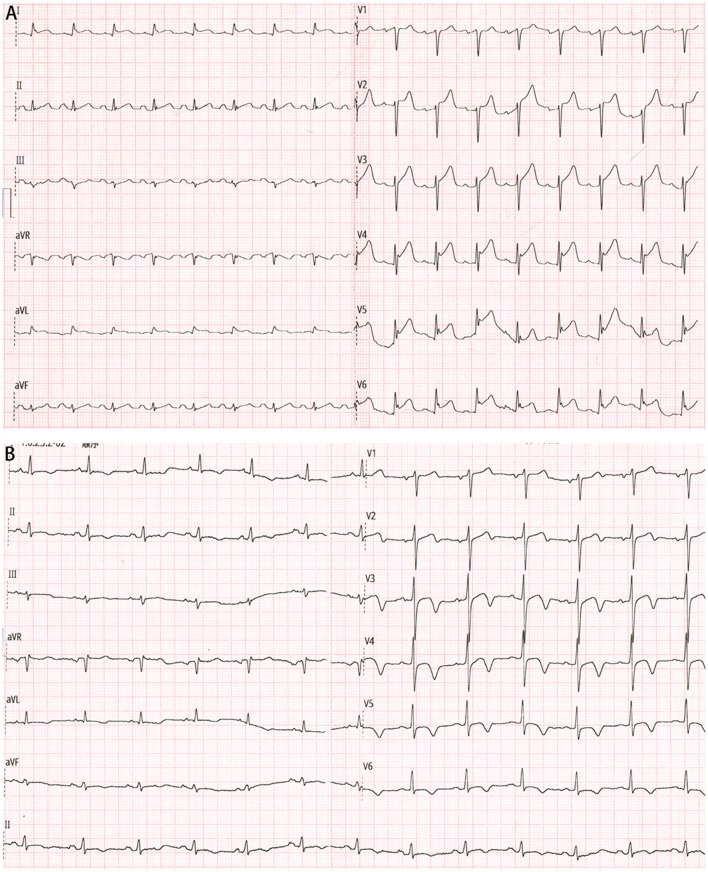
**(A)** Electrocardiogram on admission showing ST-segment elevation in the precordial leads. **(B)** Electrocardiogram after percutaneous coronary intervention showing significantly improved ST-segment elevation resolution.

A 65-year-old male patient was referred to our hospital with chest pain, palpitation, dyspnea, sweating, nausea, and vomiting. He denied smoking history and any past medical history. On arrival, he was found to be in circulatory shock, with a blood pressure of 62/47 mmHg and a heart rate of 111 beats per minute, and moist rales were audible in the lower lung fields. His electrocardiogram showed ST-segment elevation in the precordial leads (Figure 1A). Biochemical tests suggested significantly elevated high-sensitivity cardiac troponin T (1,011 ng/L). A diagnosis of STEMI was suspected.

**Figure 2:**
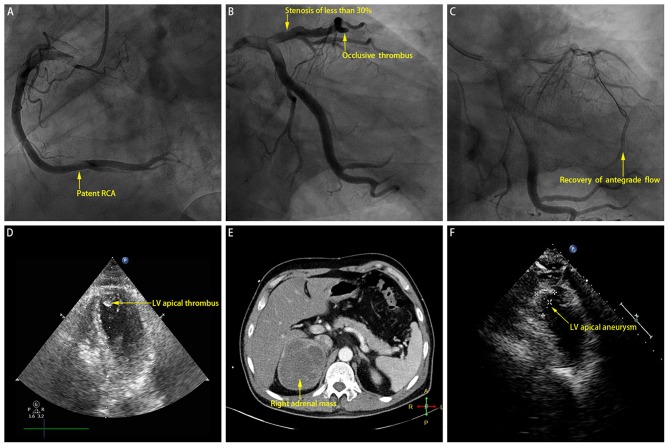
**(A)** Coronary angiogram revealing patent right coronary artery (RCA). **(B)** Coronary angiogram revealing minimal stenosis of <30% near proximal left anterior descending coronary artery (LAD) and occlusive thrombus in distal LAD. **(C)** Recovery of antegrade flow into distal LAD after manual thrombus aspiration. **(D)** Index echocardiogram revealing left ventricular (LV) apical thrombus. **(E)** Computed tomography scan revealing right adrenal mass which is of soft tissue attenuation with heterogeneous contrast enhancement. **(F)** Echocardiogram performed at the 2-month follow-up revealing left ventricular (LV) apical aneurysm.

The patient was given norepinephrine to improve hemodynamic state and vital organ perfusion. As per standard protocol, he underwent an emergency coronary angiogram (Figures 2A,B), which revealed high thrombus burden in the distal left anterior descending coronary artery (LAD) (Thrombolysis In Myocardial Infarction (TIMI) grade 0 flow), slow flow phenomenon in all other segments of the epicardial coronary arteries (TIMI grade 2 flow), and a minimal stenosis of <30% in the proximal LAD. Manual thrombus aspiration was performed repeatedly, with the intracoronary injection of glycoprotein IIb/IIIa inhibitor (tirofiban) and calcium channel blocker (diltiazem). After the procedure, the flow of the distal LAD achieved TIMI grade 2, and the lumen size recovered to the normal level (Figure 2C).

Then the patient was admitted to our cardiac intensive care unit. Vasopressor was continued to maintain blood pressure. Additional laboratory test suggested mildly increased hemoglobin A1c level (6.3%) and normal cholesterol values (TC, 4.2 mmol/L; LDL-C, 1.76 mmol/L). Few hours later, another electrocardiogram showed significantly improved ST-segment elevation resolution (Figure 1B), and the index echocardiogram (Figure 2D) showed the hypokinesis of LV apical wall which was consistent with the territory supplied by the distal LAD, left ventricular (LV) apical thrombus (13 mm × 13 mm), thickened LV septum (12–14 mm), and a borderline LV ejection fraction (50%). Anticoagulation with enoxaparin bridging to warfarin was initiated in addition to dual antiplatelet therapy.

However, the patient's condition was not further improved after initial treatment. He experienced an extremely abnormal fluctuation of blood pressure, ranging from 264/172 to 55/32 mmHg, with a sinus rate of 184–90 beats per minute. Phentolamine, an alpha-adrenergic antagonist which may be the most specific intervention in this setting, was infused to control significantly increased blood pressure under close monitoring. Hypotension was treated with fluid resuscitation administration and then intravenous norepinephrine if necessary. Further diagnostic tests were performed. Hormone assays revealed strikingly increased plasma metanephrine (10.14 nmol/L; upper reference limit: 0.50 nmol/L) and normetanephrine (11.94 nmol/L; upper reference limit: 0.90 nmol/L). Contrast-enhanced computed tomography scan showed a large mass (9.4 cm × 7.3 cm × 8.8 cm) arising from the right adrenal gland which is of soft tissue attenuation with heterogeneous contrast enhancement (Figure 2E). Accordingly, a clinical diagnosis of pheochromocytoma crisis was made.

Seven days later, the patient's blood pressure tended to be fairly steady. Oral phenoxybenzamine and metoprolol were bridged to infused vasoactive agents. He underwent thrombophilia screening studies (including antiphospholipid antibodies, protein S or C deficiency, factor V Leiden mutation, prothrombin gene mutation, antithrombin III deficiency, and elevated factor VIII), without any positive results. Then the patient was discharged with oral medications. At the 2-month follow-up, his blood pressure was well-controlled; and the repeated echocardiogram showed the disappearance of LV thrombus, persistent hypokinesis of the LV apical wall, and medium-sized LV apical aneurysm (23 mm × 14 mm; Figure 2F). The patient was referred to undergo right adrenalectomy, and the histopathological analysis confirmed the final diagnosis of pheochromocytoma.

## Discussion

This patient was diagnosed with pheochromocytoma crisis and STEMI. Based on his clinical characteristics and possible pathophysiological consequences of pheochromocytoma, spontaneous coronary thrombosis was considered the primary cause of STEMI. To our best knowledge, this is the first report regarding such a rare condition.

Previous studies have reported that some patients with pheochromocytoma may present with STEMI. However, almost all of the STEMI diagnosis in these studies were made according to the criteria of biomarker, ischemia symptom, electrocardiographic changes, and/or cardiac imaging, except one involving pathological findings (4, 7). It should be noted that all the mentioned criteria except pathological findings are less specific for differentiating ischemic myocardial injury from non-ischemic myocardial injury which may be found in myocarditis, Takotsubo syndrome, critically systemic conditions, etc. (8), and the angiographic evidence of occlusive thrombus can be used to confirm ischemic injury. Nevertheless, no study has found thrombus evidence besides widely patent coronary arteries or bystander mild lesions. Moreover, the confusion of diagnosis resulting from the similar features of STEMI with that of Takotsubo cardiomyopathy may exist in these studies. In this patient, we directly obtained the evidence of occlusive thrombus during the angiography, and all his other characteristics also fulfilled the universal definition of STEMI.

Another unique characteristic of this patient is the spontaneous thrombosis in the infarct-related artery. A few studies have depicted the association between spontaneous thrombosis and pheochromocytoma; however, no one has reported this phenomenon in the coronary arteries of patients with pheochromocytoma. The underlying mechanisms contributing to spontaneous thrombosis in this patient may be complex. First, excessively secreted catecholamines may increase the levels of factor VIII and VWF antigen and activate platelets, resulting in hypercoagulability (5). Second, the inflammatory cytokines and procoagulants secreted by tumor cells, such as the plasminogen activator inhibitor-1 which is stored in catecholamine storage vesicles and co-released with catecholamines, may play a crucial role in inducing hypercoagulability (9, 10). Third, the coronary slow flow, possibly caused by microvascular dysfunction following vasospasm due to aberrant catecholamines secretion (11, 12), may also participate in this pathologic process.

However, several potential limitations should be noted. First, we cannot completely exclude that plaque disruption of the minimal lesion located at the proximal LAD was the cause of the thrombotic event occurring in the distal LAD due to the lack of intracoronary imaging. However, the possibility should be very low according to its angiographic characteristics. Second, LV thrombus formation should be the complication of hypercoagulability and consequent STEMI, but the delay of echocardiogram seemed to make us unable to obtain more robust evidence to confirm this point. Third, we did not further perform left ventriculography during the emergency procedure because of hemodynamic instability and the presence of culprit lesion; therefore, we did not know whether this patient was also complicated by Takotsubo cardiomyopathy, a form of acute catecholaminergic myocardial stunning, whose typical imaging characteristic is the circumferential dysfunction of the involved ventricular segments.

In summary, this is an interesting and unique case. It raises awareness of the possibility of spontaneous coronary thrombosis and consequent STEMI in patients with pheochromocytoma.

## Ethics Statement

The studies involving human participants were reviewed and approved by Biomedical Research Ethics Committee, West China Hospital of Sichuan University. The patients/participants provided their written informed consent to participate in this study.

## Author Contributions

FC and MZ collected the clinical data and wrote the manuscript. XL managed the case and collected the clinical data. All the work was performed under the instructions of YP and MC.

### Conflict of Interest

The authors declare that the research was conducted in the absence of any commercial or financial relationships that could be construed as a potential conflict of interest.
